# Canthin-6-One Inhibits Developmental and Tumour-Associated Angiogenesis in Zebrafish

**DOI:** 10.3390/ph17010108

**Published:** 2024-01-12

**Authors:** Mei Fong Ng, Juliana Da Silva Viana, Pei Jean Tan, Denver D. Britto, Sy Bing Choi, Sakurako Kobayashi, Norazwana Samat, Dedrick Soon Seng Song, Satoshi Ogawa, Ishwar S. Parhar, Jonathan W. Astin, Benjamin M. Hogan, Vyomesh Patel, Kazuhide S. Okuda

**Affiliations:** 1 Cancer Research Malaysia, Subang Jaya 47500, Selangor, Malaysia; mfong.ng@gmail.com (M.F.N.); tanpeijean@gmail.com (P.J.T.); norazwana5656@gmail.com (N.S.); songdedrick@gmail.com (D.S.S.S.); vpatel.edit@gmail.com (V.P.); 2 Organogenesis and Cancer Program, Peter MacCallum Cancer Centre, Melbourne, VIC 3000, Australia; juliana.dasilvaviana@petermac.org (J.D.S.V.); sakurako.kobayashi@petermac.org (S.K.); ben.hogan@petermac.org (B.M.H.); 3 Sir Peter MacCallum Department of Oncology, University of Melbourne, Melbourne, VIC 3000, Australia; 4 Department of Molecular Medicine & Pathology, School of Medical Sciences, The University of Auckland, Auckland 1010, New Zealand; denverdesb@gmail.com (D.D.B.); j.astin@auckland.ac.nz (J.W.A.); 5 Department of Biotechnology, Faculty of Applied Sciences, UCSI University, Cheras 56000, Kuala Lumpur, Malaysia; choisb@ucsiuniversity.edu.my; 6 Brain Research Institute, School of Medicine and Health Sciences, Monash University Malaysia, Bandar Sunway 47500, Selangor, Malaysia; satoshi.ogawa@monash.edu (S.O.); ishwarparhar@gmail.com (I.S.P.); 7 Department of Anatomy and Physiology, University of Melbourne, Melbourne, VIC 3000, Australia; 8 Division of Genomics of Development and Disease, Institute for Molecular Bioscience, The University of Queensland, St. Lucia, QLD 4072, Australia; 9 Department of Biochemistry and Chemistry, La Trobe Institute for Molecular Science, La Trobe University, Bundoora, VIC 3086, Australia; 10 Centre for Cardiovascular Biology and Disease Research, School of Agriculture, Biomedicine and Environment, La Trobe University, Bundoora, VIC 3086, Australia

**Keywords:** angiogenesis, zebrafish, cancer, canthin-6-one, natural product

## Abstract

Tumour-associated angiogenesis play key roles in tumour growth and cancer metastasis. Consequently, several anti-angiogenic drugs such as sunitinib and axitinib have been approved for use as anti-cancer therapies. However, the majority of these drugs target the vascular endothelial growth factor A (VEGFA)/VEGF receptor 2 (VEGFR2) pathway and have shown mixed outcome, largely due to development of resistances and increased tumour aggressiveness. In this study, we used the zebrafish model to screen for novel anti-angiogenic molecules from a library of compounds derived from natural products. From this, we identified canthin-6-one, an indole alkaloid, which inhibited zebrafish intersegmental vessel (ISV) and sub-intestinal vessel development. Further characterisation revealed that treatment of canthin-6-one reduced ISV endothelial cell number and inhibited proliferation of human umbilical vein endothelial cells (HUVECs), suggesting that canthin-6-one inhibits endothelial cell proliferation. Of note, canthin-6-one did not inhibit VEGFA-induced phosphorylation of VEGFR2 in HUVECs and downstream phosphorylation of extracellular signal-regulated kinase (Erk) in leading ISV endothelial cells in zebrafish, suggesting that canthin-6-one inhibits angiogenesis independent of the VEGFA/VEGFR2 pathway. Importantly, we found that canthin-6-one impairs tumour-associated angiogenesis in a zebrafish B16F10 melanoma cell xenograft model and synergises with VEGFR inhibitor sunitinib malate to inhibit developmental angiogenesis. In summary, we showed that canthin-6-one exhibits anti-angiogenic properties in both developmental and pathological contexts in zebrafish, independent of the VEGFA/VEGFR2 pathway and demonstrate that canthin-6-one may hold value for further development as a novel anti-angiogenic drug.

## 1. Introduction

Angiogenesis is a process where new blood vessels form from pre-existing vasculature. Under physiological circumstances, angiogenesis is only triggered when there is a wound healing process, inflammation, or endometrial growth during the menstrual cycle [1]. However, aberrant angiogenesis is associated in pathological conditions such as cancer, chronic inflammatory diseases, ocular diseases, skin disorders, and vascular anomalies [2,3,4]. With cancer, tumour-associated angiogenesis plays a crucial role in cancer progression and metastasis as new blood vessels that are formed supply oxygen and nutrients for proliferating cancer cells and provide conduits for intravasated cancer cells to disseminate [4,5]. Cancer cells have been shown to actively secrete pro-angiogenic growth factors such as vascular endothelial growth factor A (VEGFA) to promote tumour-associated angiogenesis [6]. Consequently, small molecule tyrosine kinase inhibitors that target the VEGFA/VEGFR2 pathway have been developed such as sunitinib, sorafenib, and axitinib that are now FDA-approved therapies for multiple advanced staged cancers such as metastatic colorectal cancer and metastatic non-small-cell lung cancer [7,8]. Although anti-angiogenic drugs are effective, cancer patients treated with VEGFA/VEGFR2 inhibitors are liable to develop resistance likely due to cancer cells finding alternative means to drive tumour-associated angiogenesis (non-VEGFR pathway-driven angiogenesis) [7,8]. Additionally, studies have shown that VEGFA/VEGFR2 inhibitors can lead to increased aggressiveness of tumours [9,10,11,12]. These limitations emphasise the need for identifying novel anti-angiogenic drugs that target non-VEGFR pathways to use as monotherapy or in combination with existing VEGFA/VEGFR2 inhibitors.

Zebrafish is an attractive in vivo model for drug screening due to its low cost of maintenance, rapid developmental speed and high fecundity [13]. Approximately 70% of human protein-coding genes have zebrafish orthologues and ~82% of human disease-related genes have zebrafish counterparts, suggesting that molecular mechanisms involved in human physiopathological processes are conserved in zebrafish [14]. Of note, zebrafish embryos are optically transparent and therefore, development of both blood and lymphatic vessels can be readily visualised in real-time using zebrafish transgenics with fluorescently labelled vasculatures [13]. Specifically, development of intersegmental vessels (ISVs) and the sub-intestinal vessel (SIV) have been characterised extensively and have been shown to be dependent on angiogenic processes [15,16,17,18,19]. ISVs emerge from the dorsal aorta at approximately 20 h post-fertilisation (hpf) and grow dorsally along somites, while SIVs originate from the posterior cardinal vein at approximately 32 hpf and surrounds the developing intestine [15,16,17,18,19]. Both human and mouse cancer cell lines can be transplanted into zebrafish embryos and adult fish and these cells can be monitored for endpoints of proliferation, tumour dissemination and tumour-associated angiogenesis [20,21,22,23]. Taking advantage of these unique features of zebrafish, several anti-angiogenic agents have been identified using various zebrafish models [13].

In this current study, we used the zebrafish model to identify novel anti-angiogenic agents from our library of natural compounds isolated from Malaysian terrestrial plants (Brucea and Blumea genus) and marine species (soft coral and liverworts), as well as semi-synthetic compounds broadly consisting of curcumin derivatives, diarylpentanoids, flavonoids, and alkaloids [24,25]. To assess the anti-angiogenic potential of these compounds, we examined their ability in perturbing the development of ISVs in zebrafish embryos at 20 µM concentration. From this, we identified canthin-6-one, an alkaloid that we isolated from *Brucea javanica* (L.) Merr with anti-cancer properties [24] as a promising anti-angiogenic compound. Canthin-6-one is also present in other plants including Rutaceae, Simaroubacea, Amaranthaceae, Caryophyllacaea, Zygophyllacaea families, and fungi and marine organisms [26]. Various biological activities have been reported for canthin-6-one including anti-microbial, anti-inflammatory, and anti-tumoral activities [26,27]. However, potential anti-angiogenic properties of canthin-6-one have not been investigated in detail. We found that canthin-6-one inhibits both ISV and SIV development at 20 µM. We also demonstrated that in in vitro and in vivo, canthin-6-one can inhibit angiogenesis by impairing endothelial cell proliferation without inhibiting VEGFA-induced phosphorylation of VEGFR2 or its downstream target extracellular signal-regulated kinase (Erk). Finally, we revealed that canthin-6-one inhibits tumour-associated angiogenesis in a zebrafish B16F10 melanoma cell xenograft model and synergises with VEGFR inhibitor sunitinib malate (SM), highlighting its potential utility as a novel anti-angiogenic drug for human cancer.

## 2. Results

### 2.1. Canthin-6-One Inhibits Angiogenesis in Zebrafish

**Figure 1:**
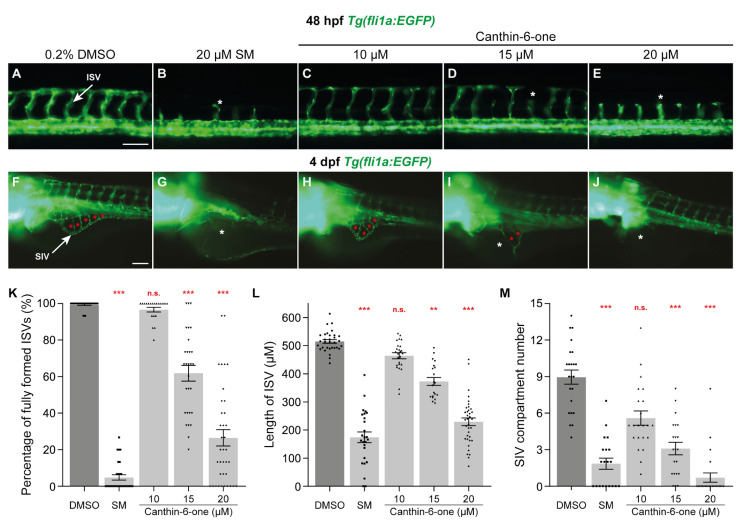
Canthin-6-one inhibits intersegmental vessel and sub-intestinal vessel development in zebrafish. (**A**–**J**) Lateral fluorescent images of 48 hpf (**A**–**E**) and 4 dpf (**F**–**J**) *Tg(fli1a:EGFP)* embryos treated with either 0.2% DMSO (**A**,**F**), 20 µM sunitinib malate (SM, **B**,**G**), or canthin-6-one at 10 µM (**C**,**H**), 15 µM (**D**,**I**), or 20 µM (**E**,**J**). White arrows indicate the intersegmental vessel (ISV, **A**) or the sub-intestinal vessel (SIV, **F**). Red asterisks indicate the vascular compartments within the SIV (**F**,**H**,**I**). White asterisks indicate the reduced/absence of ISVs (**B**,**D**,**E**) or SIV (**G**,**I**,**J**). (**K**) Quantification of the number of fully formed ISVs in 48 hpf *Tg(fli1a:EGFP)* embryos treated with either 0.2% DMSO (*n* = 30 embryos), 20 µM SM (*n* = 29 embryos), or canthin-6-one at 10 µM (*n* = 20 embryos), 15 µM (*n* = 29 embryos), or 20 µM (*n* = 37 embryos). (**L**) Quantification of the length of ISVs in 48 hpf *Tg(fli1a:EGFP)* embryos treated with either 0.2% DMSO (*n* = 32 embryos), 20 µM SM (*n* = 26 embryos), or canthin-6-one at 10 µM (*n* = 25 embryos), 15 µM (*n* = 20 embryos), or 20 µM (*n* = 37 embryos). (**M**) Quantification of the number of SIV compartments in 4 dpf *Tg(fli1a:EGFP)* larvae treated with either 0.2% DMSO (*n* = 23 larvae), 20 µM SM (*n* = 20 larvae), or canthin-6-one at 10 µM (*n* = 22 embryos), 15 µM (*n* = 21 embryos), or 20 µM (*n* = 24 embryos). Statistical test: Kruskal-Wallis test was conducted for graphs (**K**–**M**). *p* ≤ 0.001 (***), *p* ≤ 0.01 (**), *p* > 0.05 (not significant, n.s.). Scale bars: 100 µm.

In an effort to identify novel anti-angiogenic compounds from our in-house library of 70 natural compounds isolated from plants and marine species and 137 semi-synthetic compounds [24,25], we took advantage of the *Tg(fli1a:EGFP)* transgenic zebrafish, where the blood vessels are fluorescently labelled [28]. The embryos were treated at 16 hpf, just prior to primary sprouting of ISVs [18], to examine the effect of the test compounds on ISV development. An initial concentration of 20 µM was chosen as a starting concentration as this was the concentration required for SM, a pan-VEGFR kinase inhibitor to completely inhibit ISV development in zebrafish (Figure 1B and Figure S1A–D) [13]. Overall, we identified 3 compounds from our library, including kaempferol, which had previously been shown to be anti-angiogenic [29], that inhibited ISV development at 48 hpf (Figure S1E–L). Of these, canthin-6-one, an alkaloid isolated from a plant named *Brucea javanica* (L.) Merr [24], was noted to have minimal developmental phenotypic defects such as body shortening and developmental delay, and therefore was chosen for further characterisation (Figure S1E’–G’). As the amount of isolated canthin-6-one was limited in our library, we purchased commercially available canthin-6-one for all subsequent experiments. We next sought to identify the optimal concentration required to confer this anti-angiogenic activity in zebrafish without inducing off-target effects. For this, we treated *Tg(fli1a:EGFP)* embryos with canthin-6-one at concentrations ranging from 10–40 µM. As previously shown, treatment with 20 µM SM reduced the number of fully formed ISVs as well as ISV length, when compared to embryos treated with 0.2% dimethyl sulfoxide (DMSO, Figure 1A,B,K,L) [13]. We observed that canthin-6-one attenuated ISV formation in a dose dependent manner, where the number of fully formed ISVs and ISV length was reduced in embryos treated with both 15 and 20 µM canthin-6-one but not for 10 µM (Figure 1A,C–E,K,L). Higher concentrations of canthin-6-one (30 and 40 µM) resulted in moderate to severe body length shortening and body curvature of embryos, and thus were not included in our analysis (Figure S1M–O’). To exclude the possibility that canthin-6-one only inhibits trunk vascular development, we investigated whether canthin-6-one inhibits the development of SIVs. For this, we treated the embryos with canthin-6-one at 24 hpf (after ISVs have sprouted and before SIV development [17,18]) and measured SIV development at 4 days post-fertilisation (dpf), by quantifying the number of vessel chambers within the SIV. As anticipated, treatment with 20 µM SM significantly reduced the number of SIV compartments when compared to 0.2% DMSO treated larvae (Figure 1F,G,M). We also noted that canthin-6-one inhibited the formation of SIVs in a dose dependent manner with reduction in the number of SIV vessel compartments observed at both 15 and 20 µM, while 10 µM did not significantly inhibit SIV development (Figure 1F,H–J,M). Collectively, we demonstrated that canthin-6-one at 20 µM is anti-angiogenic in developing zebrafish and this concentration was used for all subsequent experiments.

### 2.2. Canthin-6-One Inhibits Endothelial Cell Proliferation

**Figure 2:**
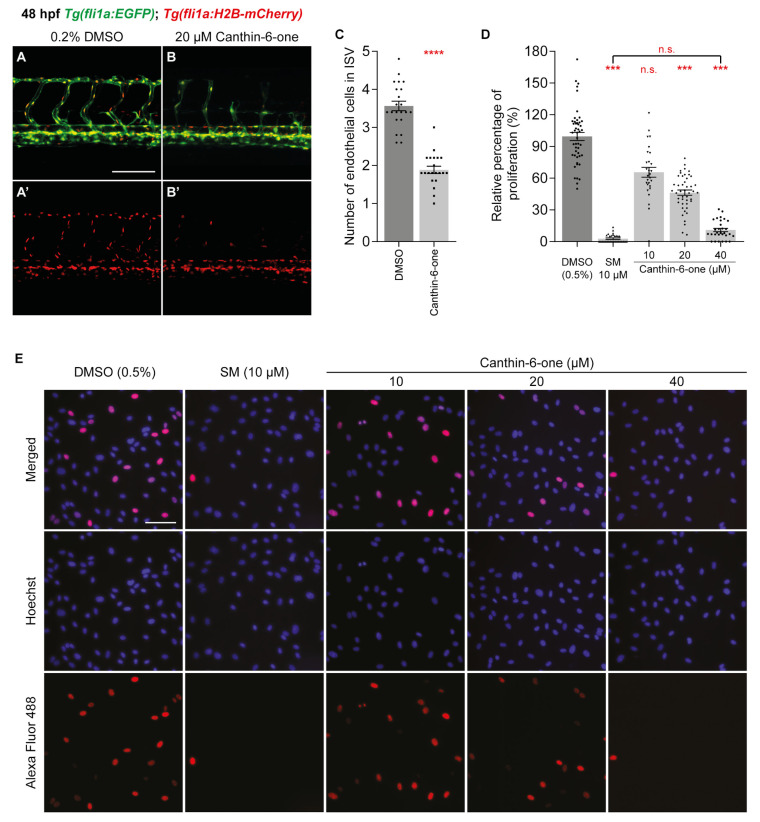
Canthin-6-one inhibits endothelial cell proliferation in zebrafish and HUVECs. (**A**,**B’**) Lateral confocal images of 48 hpf *Tg(fli1a:EGFP)*;*Tg(fli1a:H2B-mCherry)* embryos treated with either 0.2% DMSO (**A**,**A’**) or 20 µM canthin-6-one (**B**,**B’**). Images (**A’**,**B’**) represent the *fli1a:H2B-mCherry* expression of images (**A**,**B**). (**C**) Quantification of intersegmental vessel (ISV) endothelial cell number in 48 hpf *Tg(fli1a:EGFP)*;*Tg(fli1a:H2B-mCherry)* embryos treated with either 0.2% DMSO (*n* = 22 embryos) or 20 µM canthin-6-one (*n* = 21 embryos). Each data points represent an average of 5 ISVs from one embryo. (**D**) Quantification of relative percentage of proliferating HUVECs upon treatment with either 0.2% DMSO (*n* = 48 views), 10 µM sunitnib malate (SM, *n* = 30 views), or canthin-6-one at 10 µM (*n* = 28 views), 20 µM (*n* = 46 views), or 40 µM (*n* = 30 views). Results from at least 3 independent replicates. (**E**) Representative images of HUVECs treated with either 0.2% DMSO, 10 µM SM, or canthin-6-one at indicated concentrations. Cells were stained with Hoechst 33,342 (blue) and Alexa Fluor 488 (red) using the Click-iT assay. Blue indicates viable cells and red indicates proliferating cells. Statistical test: Mann-Whitney test for (**C**) and Kruskal-Wallis test for (**D**). *p* ≤ 0.0001 (****), *p* ≤ 0.001 (***), *p* > 0.05 (not significant, n.s.). Scale bars: 100 µm (**A**), 250 µm (**E**).

We have previously shown that canthin-6-one is able to inhibit proliferation of human cancer cell lines PC-3 and HeLa [24]. As endothelial cell proliferation is critical in angiogenesis, we examined if canthin-6-one could impact endothelial cell proliferation in ISVs. To assess this, we treated 16 hpf *Tg(fli1a:EGFP)*;*Tg(fli1a:H2B-mCherry)* transgenic embryos with 20 µM canthin-6-one and quantified endothelial cell number (mCherry-positive endothelial cell nuclei) of ISVs at 48 hpf. Treatment of 20 µM canthin-6-one significantly reduced the number of endothelial cell nuclei in ISVs when compared to 0.2% DMSO treated embryos, indicating that the reduced ISV development observed in embryos treated with canthin-6-one is due to reduced endothelial cell proliferation (Figure 2A–C). To obtain further evidence, we investigated whether canthin-6-one is able to reduce proliferation of human umbilical vein endothelial cells (HUVECs) using the Click-iT assay [25]. Prior to the Click-iT assay, we performed a cell viability assay using both SM and canthin-6-one to determine the most suitable concentration to use in our in vitro experiments. We assessed that the IC_50_ of canthin-6-one in HUVECs was ~20 µM. and thus we chose a concentration range of 10–40 µM for our subsequent analysis (Figure S2A–D). Consistent with our observations in zebrafish, canthin-6-one inhibited endothelial cell proliferation in a dose dependent manner, with significant inhibition of endothelial cell proliferation observed in HUVECs treated with 20 and 40 µM canthin-6-one (Figure 2D,E). We have previously shown that canthin-6-one inhibits proliferation of human PC-3 and HeLa cancer cells by inducing G2/M cell cycle block [24]. Similarly, our data show a marginal increase in the cell population in G2 phase in HUVECs treated with SM or canthin-6-one at 10–40 µM, suggesting a G2/M block in HUVECs treated with canthin-6-one (Figure S2E). To exclude the possibility that canthin-6-one is inducing endothelial cell apoptosis, we measured cleaved caspase 3 levels and found no elevated cleaved caspase 3 levels in HUVECs treated with 10–40 µM canthin-6-one (Figures S3 and S4). Collectively, both our in vitro and in vivo analysis suggested that canthin-6-one exerts its anti-angiogenic activity by inhibiting endothelial cell proliferation.

### 2.3. Canthin-6-One Does Not Inhibit VEGFA/VEGFR2/ERK Signalling

**Figure 3:**
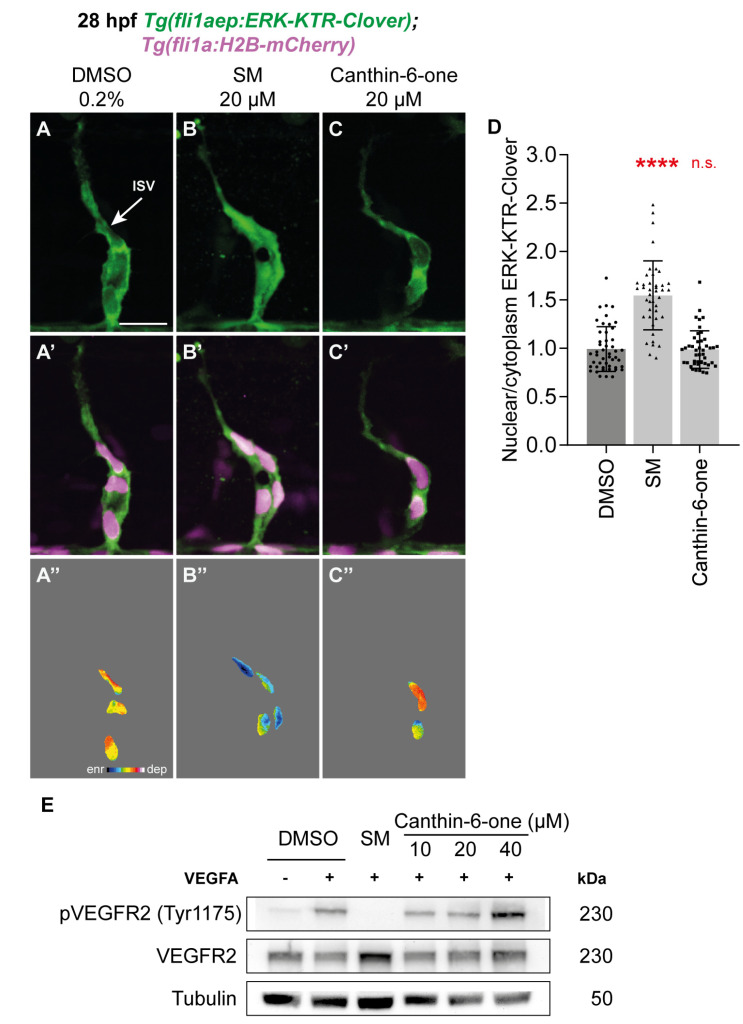
Canthin-6-one does not inhibit the VEGFA/VEGFR2 pathway. (**A**–**C’’**) Lateral spinning disc confocal images of intersegmental vessel (ISV) endothelial cells in 28 hpf *Tg(fli1aep:ERK-KTR-Clover)*;*Tg(fli1a:H2B-mCherry)* embryos treated with either 0.2% DMSO (**A**,**A’**,**A’’**), 20 µM SM (**B**,**B’**,**B’’**), or 20 µM canthin-6-one (**C**,**C’**,**C’’**). Images (**A**–**C**) show *fli1aep:ERK-KTR-Clover* expression, while images (**A’**–**C’**) show both *fli1aep:ERK-KTR-Clover* and *fli1a:H2B-mCherry* expression. Images (**A’’**–**C’’**) show the nuclear *fli1aep:ERK-KTR-Clover* expression with intensity differences represented in 16 colour LUT (Fiji). The *fli1a:H2B-mCherry* signal was used to mark the nucleus. (**D**) Quantification of nucleus/cytoplasm ERK-KTR-Clover intensity in leading ISV endothelial cells of 28 hpf embryos treated with either 0.2% DMSO (*n* = 48 ISV endothelial cells from 10 embryos), 20 µM SM (*n* = 42 ISV endothelial cells from 10 embryos), or 20 µM canthin-6-one (*n* = 45 ISV endothelial cells from 12 embryos). (**E**) Western blot analysis of lysates isolated from HUVECs treated with either 0.2% DMSO, 10 µM sunitinib malate (SM), or canthin-6-one at indicated concentrations for 1 h and stimulated with vascular endothelial growth factor A (VEGFA) for 10 min (*n* = 3). Protein levels of pVEGFR2, total VEGFR2, or Tubulin were assessed. The full-length blots are presented in Figure S6. Statistical test: Kruskal-Wallis test was conducted for graph (**E**). *p* ≤ 0.0001 (****), *p* > 0.05 (not significant, n.s.). Scale bar: 50 µm (**A**).

The VEGFA/VEGFR2 pathway is essential for angiogenesis in both zebrafish and mammals [6,30]. VEGFA regulates angiogenesis by binding to VEGFR2 and this results in the phosphorylation of VEGFR2 and its downstream targets such as ERK [6,30]. To investigate whether canthin-6-one is able to inhibit Vegfa/Vegfr2-mediated Erk phosphorylation in endothelial cells in vivo, we treated 26 hpf *Tg(fli1aep:ERK-kinase translocation reporter (KTR)-Clover)* embryos with 20 µM canthin-6-one for 2 h and quantified Erk activity in leading endothelial cells in sprouting ISVs, which has high Vegfa/Vegfr2/Erk activity [30,31,32]. The *Tg(fli1aep:ERK-KTR-Clover)* transgenic line takes advantage of a fluorescent-based kinase activity reporter of Erk activity to allow dynamic endothelial cell Erk activity to be quantified in vivo [32,33]. As previously reported, leading endothelial cells of ISVs in 0.2% DMSO treated embryos had high Erk activity (nuclear depleted ERK-KTR-Clover expression), while this Erk activity was impaired in embryos treated with 20 µM SM (nuclear enriched ERK-KTR-Clover expression) (Figure 3A,B’’,D) [32]. The Erk activity remained high in leading endothelial cells of ISVs in embryos treated with 20 µM canthin-6-one, comparable to 0.2% DMSO treated embryos (Figure 3C,D). This indicates that canthin-6-one does not inhibit Vegfa/Vegfr2/Erk activity in ISV endothelial cells to inhibit angiogenesis. Next, we investigated if canthin-6-one inhibits VEGFA-induced VEGFR2 phosphorylation in HUVECs using western blot analysis. SM treatment inhibited VEGFA-induced phosphorylation of VEGFR2 in HUVECs but canthin-6-one treatment at 10–40 µM did not (Figure 3E, Figures S5A and S6). We also performed a molecular docking analysis which further complemented our findings from above. Docking of SM on VEGFR2 exhibited a binding affinity of -9.28 kcal/mol of free energy of binding while erlotinib (EGFR inhibitor) produced a less favourable binding affinity of −7.36 kcal/mol of free energy of binding (Figure S7A,B,D). In comparison, the binding affinity of canthin-6-one on VEGFR2 was lower than erlotinib at −7.1 kcal/mol of free energy of binding, reflecting weaker binding affinity to VEGFR2 (Figure S7A,C). Finally, we investigated whether canthin-6-one inhibits gene expression of *vegfaa*, *vegfab* (zebrafish orthologues of VEGFA), *kdr*, and *kdrl* (zebrafish orthologues of VEGFR2) in zebrafish using qPCR analysis. Neither SM nor canthin-6-one treatment at 16 hpf attenuated the mRNA levels of *vegfaa*, *vegfab* and *kdr* in 48 hpf embryos (Figure S5B). The mRNA level of *kdrl* was significantly reduced in zebrafish embryos treated with 20 µM SM (reduced by ~38.58%) but not in embryos treated with 20 µM canthin-6-one (reduced by ~17.7%, not significant, Figure S5B). Collectively, our data indicated that unlike SM, canthin-6-one does not impair angiogenesis by inhibiting VEGFA/VEGFR2/ERK signalling activity.

### 2.4. Canthin-6-One Inhibits Tumour-associated Angiogenesis and Synergises with SM

**Figure 4:**
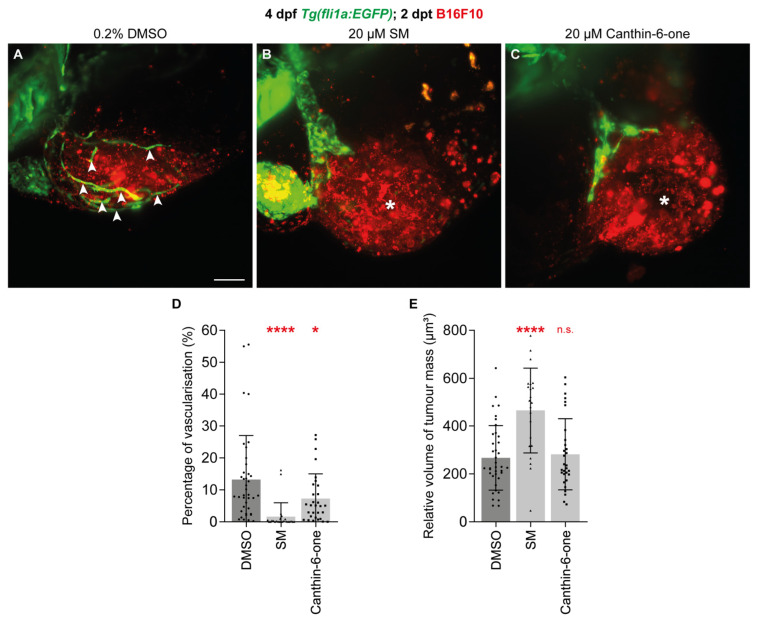
Canthin-6-one inhibits tumour-associated angiogenesis in zebrafish. (**A**–**C**) Lateral light-sheet microscope images of 2 dpt (4 dpf) *Tg(fli1a:EGFP)* larvae injected with B16F10 melanoma cells and treated with either 0.2% DMSO (**A**), 20 µM sunitinib malate (SM, **B**), or 20 µM canthin-6-one (**C**) from 2 dpf. White arrow heads show tumour-associated blood vessels and asterisks indicate the absence of tumour-associated angiogenesis. (**D,E**) Quantification of blood vessels within the B16F10 tumour mass relative to tumour mass volume (**D**) or tumour mass volume (**E**) in 2 dpt (4 dpf) *Tg(fli1a:EGFP)* larvae injected with B16F10 melanoma cells and treated with either 0.2% DMSO (*n* = 38 larvae), 20 µM SM (*n* = 23 larvae), or 20 µM canthin-6-one (*n* = 30 larvae). Statistical test: Ordinary one-way ANOVA test was conducted for graphs (**D**,**E**). *p* ≤ 0.0001 (****), *p* ≤ 0.05 (*), *p* > 0.05 (not significant, n.s.). Scale bar: 100 µm (**A**).

Tumour-associated angiogenesis is essential for tumour growth and progression [5,34]. As canthin-6-one inhibits developmental angiogenesis, we investigated if canthin-6-one could also impact tumour-associated angiogenesis. To test this, we took advantage of the zebrafish B16F10 melanoma cell xenotransplantation model, which we have previously shown to be associated with robust tumour-associated angiogenesis [20]. As previously shown, xenotransplantation of B16F10 cells stimulated angiogenesis from the common cardinal vein to within the B16F10 tumour mass in the zebrafish larvae after 2 days post-transplantation (dpt, 4 dpf, Figure 4A white arrow heads) [20]. As expected, the blood vessel volume within the B16F10 tumour mass was lower in 2 dpt larvae treated with 20 µM SM when compared to 0.2% DMSO-treated larvae (Figure 4A,B,D). Similarly, treatment with 20 µM canthin-6-one inhibited blood vessel growth within the B16F10 tumour mass in 2 dpt larvae (Figure 4A–D). Importantly, the volume of B16F10 tumour mass was unchanged in canthin-6-one treated larvae, showing that the reduced tumour-associated angiogenesis is not due to reduced tumour growth (Figure 4E).

**Figure 5:**
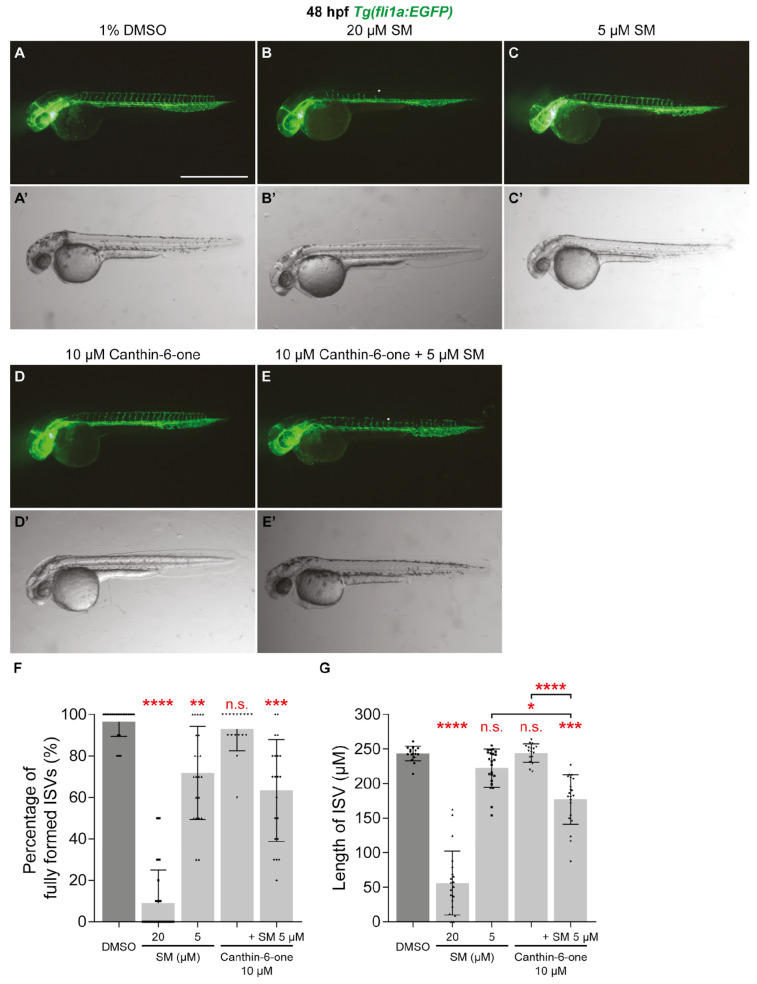
Canthin-6-one synergises with VEGFR inhibitor sunitinib malate. (**A**–**E’**) Lateral fluorescent (**A**–**E**) and trans-light images (**A**–**E’**) of 48 hpf *Tg(fli1a:EGFP)* embryos treated with either 1% DMSO (**A**,**A’**), 20 µM sunitinib malate (SM, **B**,**B’**), 5 µM SM (**C**,**C’**), 10 µM canthin-6-one (**D**,**D’**), or co-treatment of 5 µM SM and 10 µM canthin-6-one (**E**,**E’**). White asterisks indicate the absence of intersegmental vessels (ISVs). (**F**,**G**) Quantification of the percentage of fully formed ISVs (**F**) or length of ISVs (**G**) in 48 hpf *Tg(fli1a:EGFP)* embryos treated with either 1% DMSO (*n* = 23), 20 µM SM (*n* = 22), 5 µM SM (*n* = 23), 10 µM canthin-6-one (*n* = 17), or co-treatment of 5 µM SM and 10 µM canthin-6-one (*n* = 21). Statistical test: Kruskal-Wallis test was conducted for graphs (**F**,**G**). *p* ≤ 0.0001 (****), *p* ≤ 0.001 (***), *p* ≤ 0.01 (**), *p* ≤ 0.05 (*), *p* > 0.05 (not significant, n.s.). Scale bar: 1 mm (**A**).

Co-treatment strategies, particularly with non VEGFR inhibitors have been suggested as a potential approach for overcoming resistance and toxicity of VEGFR inhibitors as cancer therapeutics [6]. To investigate whether canthin-6-one and VEGFR inhibitor SM synergise, we tested if co-treatment of canthin-6-one and SM at sub-optimal doses is able to impair ISV development. Unlike 20 µM SM, treatment with 5 µM SM at 16 hpf minimally reduced the number of fully formed ISVs and did not reduce ISV length at 48 hpf (Figure 5A–C’,F,G). Similarly, treatment with 10 µM canthin-6-one at 16 hpf did not inhibit ISV development (Figure 5D,D’,F,G). While we did not see any synergistic effect on the impairment of the number of fully formed ISVs, co-treatment of 5 µM SM and 10 µM canthin-6-one at 16 hpf significantly reduced ISV length at 48 hpf when compared embryos treated with 1% DMSO, or 5 µM SM or 10 µM canthin-6-one alone (Figure 5E–G). Collectively, our results showed that canthin-6-one is able to inhibit tumour-associated angiogenesis and synergises with VEGFR inhibitor SM.

## 3. Discussion

Through an anti-angiogenic drug screen using transgenic zebrafish, we identified canthin-6-one, an indole alkaloid that we and others have shown to possess anti-cancer properties [24,26], as a promising anti-angiogenic agent. We showed that canthin-6-one is not a VEGFR inhibitor but an inhibitor of endothelial cell proliferation and synergises with the VEGFR inhibitor SM. We also revealed that canthin-6-one inhibits tumour-associated angiogenesis in vivo, highlighting the potential of canthin-6-one as a novel cancer therapy. Interestingly, Gong and colleagues showed that canthin-6-one at >200 µM did not inhibit zebrafish ISV development [35]. This is likely due to the treatment time used in their study where canthin-6-one was treated at 24 hpf, a much later time point after initiation of primary sprouting. Hence, the window for the potential inhibitory effect of canthin-6-one on primary (ISV) sprouting could have been missed. In contrary, we were able to show that canthin-6-one (20 µM) inhibits SIV development in 4 dpf larvae when treated at 24 hpf, and tumour-associated angiogenesis in 2 dpt larvae xenotransplanted with B16F10 melanoma cells, when treated at 48 hpf.

Probing further into the potential mechanism on how canthin-6-one may be conferring its anti-angiogenic activity, we investigated whether canthin-6-one inhibits the VEGFR2 pathway involved in angiogenesis. Broadly, angiogenesis is triggered by pro-angiogenic growth factors such as VEGFA, angiopoietin-1 and 2 (ANGPT1 and 2), fibroblast growth factor (FGF) and many others [3,36]. Among all, the VEGFA/VEGFR2 pathway is the major regulator for most angiogenic processes [6]. Developmental angiogenesis in zebrafish relies heavily on the Vegfa/Vegfr2 signalling, for instance, mutants of *vegfaa*, *vegfab*, and *kdrl* have reduced development of both ISVs and SIV [15,16,19,37,38,39]. Unlike VEGFR inhibitor SM however, our results indicate that canthin-6-one does not inhibit VEGFA-induced phosphorylation of VEGFR2 in HUVECs or VEGFR- dependent phosphorylation of Erk in ISV endothelial cells in vivo. This could be because canthin-6-one is targeting alternative angiogenic pathways which are important for zebrafish angiogenesis. For example, canthin-6-one may target the PI3K/AKT signalling directly, which was recently shown to be essential for ISV endothelial cell proliferation [38]. However, unlike PI3K inhibitors which only affect ISV endothelial cell proliferation and not ISV endothelial cell migration in zebrafish [38], canthin-6-one treatment appears to impair both ISV endothelial cell proliferation and migration (Figure 1E). Another possible target for canthin-6-one is the ANGPT/TIE signalling, which is required for angiogenesis in zebrafish [40] and had been suggested as a potential target for a canthin-6-one derivative 1-hydroxymethyl-8-hydroxy-β-carboline [35]. We also provided evidence that treatment of HUVECs with canthin-6-one marginally increased the cell population in G2 phase, indicating that canthin-6-one may inhibit angiogenesis by inducing endothelial cell cycle arrest at G2/M phase. Further studies, including western blot analysis of key proteins of pathways listed above (PI3K, AKT, ANGPT, TIE, cyclin-dependent kinase 1 (CDK1)), are needed to definitively elucidate the molecular targets of canthin-6-one.

Although most FDA-approved anti-angiogenic drugs for cancer treatment are VEGFA/VEGFR2 inhibitors, these inhibitors can lead to development of resistances or increased tumour aggressiveness in patients [6,7]. In contrast, only a few FDA-approved anti-angiogenic drugs, such as temsirolimus and everolimus (mTOR inhibitors), do not directly target the VEGFA/VEGFR2 pathway [7]. These non-VEGFR inhibitors can be used in combination with VEGFA/VEGFR2 inhibitors to overcome the limitations associated with VEGFA/VEGFR2 inhibitors. For example, everolimus is used to treat advanced clear cell renal cancer patients who have deteriorated after treatment with VEGFR inhibitor sorafenib or sunitinib [41,42]. As an anti-angiogenic compound that works independent of the VEGFA/VEGFR2 pathway, canthin-6-one shows promise as a novel anti-angiogenic drug, either for use as monotherapy or in combination with existing VEGFA/VEGFR2 inhibitors.

While this study provides evidence that canthin-6-one may be a promising anti-angiogenic agent, there are several key limitations. First, we show that a relatively high concentration (20 µM) of canthin-6-one was required to exert its anti-angiogenic activity. As there are many derivatives of canthin-6-one [27,35], structural activity relationship studies could be conducted as part of efficacy optimisation to identify the most optimal drug lead for further drug development. Secondly, although the synergistic activity of SM and canthin-6-one is promising, further investigation is needed to determine whether SM and canthin-6-one synergistically impair tumour-associated angiogenesis as well. Finally, the efficacy of canthin-6-one observed thus far had only been shown in zebrafish and mammalian cell culture and therefore needs to be reproduced in mammalian in vivo models to truly examine its clinical relevance.

## 4. Materials and Methods

### 4.1. Zebrafish Maintenance and Imaging

Transgenic zebrafish strains used in this study were *Tg(fli1a:EGFP)^y1^* [28], *Tg(fli1a:H2B-mCherry)^uq37bh^* [43], and *Tg(fli1aep:ERK-KTR-Clover)^uq39bh^* [32]. Larvae/embryos were anaesthetised in 0.08 mg/mL tricaine and mounted as previously described [44]. Zebrafish live-imaging was conducted using either an Olympus MVX fluorescent microscope, Zeiss LSM 710 FCS confocal microscope, Zeiss Elyra 780 confocal microscope, Nikon Yokogawa CSU-W1 spinning disc confocal microscope, or the Zeiss Z. 1 lightsheet fluorescent microscope.

### 4.2. Chemical Administration

The in-house compound library consisted of compounds isolated from natural (plants and marine organisms) and semi-synthetic compounds synthesised by our collaborating research groups from the University Malaysia Sabah and the University of Putra Malaysia. The compound library has also been used for identifying potential anti-cancer agents in separate studies [24,25]. Zebrafish embryos at 16 hpf (ISV quantification at 48 hpf), 24 hpf (SIV quantification at 4 dpf) or 48 hpf (tumour-angiogenesis quantification at 2 dpt (4 dpf)) were treated with either vehicle control (0.2–1% DMSO), sunitinib malate (SM, SelleckChem, TX, USA), or canthin-6-one (ChemFaces, Wuhan, China) in E3 medium at indicated concentrations. All compounds were dissolved in DMSO at a stock concentration of 10 mM and stored at −20 °C until use.

### 4.3. Quantification of Angiogenesis and Endothelial Erk Activity in Zebrafish

The number of fully formed ISVs (ISVs that have reached the level of the dorsal longitudinal anastomotic vessel in each somites from the 6th to the 20th somite (from the anterior side) were manually counted and represented as a percentage. The ISV length was measured from the point where the ISVs sprout from the dorsal aorta up to the tip of the ISV using the cellSens imaging software (version 1.16, Evident, Tokyo, Japan). SIV quantification was performed by manual counting of the number of vascular compartments formed within the SIVs from both sides (as shown in Figure 1F–J) as previously described [13]. Endothelial cell number in ISVs in each somite from 15th to the 20th somite were manually counted using the cell counter function in the Fiji image processing software (version 1) [45]. Erk activity in ISV endothelial cells was measured by comparing the nuclear/cytoplasm ERK-KTR-Clover intensity using a custom written ImageJ macro as previously described [32]. In Figure 3B’–D’, endothelial cell ERK-KTR-Clover intensity in nuclei is represented after masking the nuclear expresion using the H2B-mCherry and presenting the ERK-KTR-Clover intensity in 16 colour LUT (Fiji).

### 4.4. Cell Culture and Cell Labelling

HUVECs (Gibco, ThermoFisher Scientific, Waltham, MA, USA) were maintained from passages 3–8 in EndoGRO-VEGF Complete Culture Media Kit (Merck, Dermstadt, Germany) according to the manufacturer’s protocol. Mouse B16F10 melanomacells were grown and maintained in RPMI 1640 medium (Gibco, ThermoFisher Scientific, Waltham, MA, USA) supplemented with 1% *v*/*v* pen-strep and 10% *v*/*v* fetal bovine serum (FBS) according to the manufacturer’s protocol. B16F10 cells were labelled with CellTracker CM-DiI (ThermoFisher Scientific, Waltham, MA, USA) prior to zebrafish xenotransplantation. Briefly, trypsinised cells were centrifuged for 5 min at 1200 rpm and resuspended in 5 µg/mL CM-DiI in PBS, incubated for 10 min at 37 °C and an additional 20 min at 4 °C. Subsequently, B16F10 cells were centrifuged for 5 min at 1200 rpm and washed with FBS and twice with PBS. B16F10 cells were resuspended in RPMI 1640 medium with 10% GBS prior to injections into zebrafish embryos.

### 4.5. Click-iT Cell Proliferation Assay and Cell Cycle Analysis

Cell proliferation was evaluated by the incorporation of 5-ethynyl-2′deoxyuridine (Edu) using the Click-iT Edu Cell Proliferation Assay (ThermoFisher Scientific, MA, USA) as previously described [25]. Briefly, approximately 9 × 10^4^ HUVECs were seeded onto coverslips placed in a 12-well plate 24 h prior to treatment. HUVECs were treated with either 0.5% DMSO, 10 µM SM, or canthin-6-one at indicated concentrations for 24 h. At 4 h before end of treatment, Edu (10 µM) was added into each treatment well. Cells were fixed using 3.7% formaldehyde and pemeabilised using 0.5% Triton X-100 in PBS at the end of treatment. Next, cells underwent Edu conjugation to Alexa Fluor 488 and counterstained with Hoechst 33,342 according to the manufacturer’s protocol and mounted onto glass microscope slides before imaging. Cells were imaged using the Olympus IX-81 live-cell imaging system equipped with the XcellencePro imaging software (version 2.0 (build 4768), Evident, Tokyo, Japan). The nuclei of viable cells were labelled with Hoechst and proliferating cells were labelled with Alexa Fluor 488. The number of proliferating HUVECs were quantified manually and represented as percentage. Cell cycle analysis was done as previously described [24] on HUVECs treated for 24 h with either 0.5% DMSO, 10 µM SM, or canthin-6-one at indicated concentrations.

### 4.6. Western Blot and qPCR Analysis

For measuring phosphorylation levels of VEGFR2, HUVECs were serum starved for 16 h prior to treatment for 1 h with either 0.5% DMSO, 10 µM SM or canthin-6-one at indicated concentrations. Treated cells were then stimulated with 50 ng/µL of recombinant human VEGF165 (Bio-Techne, Minneapolis, MN, USA) for 10 min and cell lysates were isolated as previously described [46]. For measuring cleaved caspase 3 levels, HUVEC were treated with aforementioned treatments with an additional treatment of 500 nM of doxorubicin as a control, for 24 h. Western blot analysis was performed as previously described [46]. Primary antibodies used for western blot analysis were pVEGFR2 (#2478), VEGFR2 (#2479), β-tubulin (#2128), cleaved caspase 3 (#9664T), caspase 3 (#14220T), and GAPDH (#2118, Cell Signalling Technology, Danvers, MA, USA). Zebrafish RNA was extracted from 48 hpf embryos treated with either 0.2% DMSO, 20 µM SM, or 20 µM canthin-6-one as previously described. qPCR was performed using total RNA extracted from 48 hpf zebrafish embryos treated with either 0.2% DMSO, 20 µM SM, or 20 µM canthin-6-one from 16 hpf as previously described [25]. The following primers were used for qPCR analysis:

*actb1*-qpcr-forward 5′-GAAGGAGATCACCTCTCTTGCTC-3′

*actb1*-qpcr-reverse 5′-GTTCTGTTTAGAAGCACTTCCTGTG-3′

*vegfaa*-qpcr-forward 5′-GCTGTAAAGGCTGCCCACATAC-3′

*vegfaa*-qpcr-reverse 5′-ACCAGCAGCTCTCGGGTCTT-3′

*vegfab*-qpcr-forward 5′-AAGGACCTGCAGATGTGACAAA-3′

*vegfab*-qpcr-reverse 5′-TCCTTCATGTCCGTTCTCAAGTC-3′

*kdr*-qpcr-forward 5′-CAAGTAACTCGTTTTCTCAACCTAAGC-3′

*kdr*-qpcr-reverse 5′-GGTCTGCTACACAACGCATTATAAC-3′

*kdrl*-qpcr-forward 5′-TAATCCTGGAGAACGGAACC-3′

*kdrl*-qpcr-forward 5′-TTCAGCGTCTTCAGGTCATC-3′

### 4.7. B16F10 Xenotransplantation

B16F10 xenotransplantation was performed as previously described [20]. Briefly, approximately 300–500 CM-DiI-labelled B16F10 cells were injected into the perivitelline space of 48 hpf *Tg(fli1a:EGFP)* embryos using a Eppendorf FemtoJet 4i (Merck, Dermstadt, Germany). Only embryos with visibly injected cells within the perivitelline space were selected for treatment. One hour after injection, embryos were treated with either 0.2% DMSO, 20 µM SM, or 20 µM canthin-6-one and incubated at 35 °C for 2 days before imaging. Graft vascularisation was quantified using previously described methods with modifications [20]. Briefly, volumes of the tumour mass (CM-DiI-stained, red) and blood vessels (EGFP-labelled, green) were measured using the Volocity image analysis software (version 5.4, Quorum Technologies, Sacramento, CA, USA) within the 3D area of interest encompassing the entire graft. The volume of graft vascularisation was divided by the graft volume to measure the percentage of graft vascularisation.

### 4.8. Molecular Docking Analysis

Molecular docking simulations were conducted using the AutoDock 4.2 [47] against VEGFR2 to estimate the binding affinity of canthin-6-one. SM and erlotinib were incorporated as positive and negative controls, respectively for the analysis. X-ray structure of VEGFR2 (PDB ID: 4AGD) was obtained from the Protein Data Bank (PDB; http://www.rcsb.org/pdb/home/home.do). Ligand structures were obtained from PubChem website (http://pubchem.ncbi.nlm.nih.gov/). A total of 100 runs of independent docking simulations were generated for canthin-6-one, SM, and erlotinib. The lowest free energy of binding with the most populated cluster were selected and visualised using Visual Molecular Dynamics (VMD) software (version 1.9.3) [48].

### 4.9. Statistical Analysis

All statistical analysis was performed using Prism sofrware (GraphPad Prism, Prism 8, version 8.3.0). When comparing two groups, two-tailed Mann-Whitney test was used. Unless otherwise indicated, for multiple group comparisons, one-way analysis of variance (ANOVA) test was used for normally distributed data, and Kruskal-Wallis test was used for non-normally distributed data. Normal distribution was assessed by Shapiro-Wilk test. Stars indicate *p*-value as level of significance with *p* ≤ 0.0001 (****), *p* ≤ 0.001 (***), *p* ≤ 0.01 (**), *p* ≤ 0.05 (*), and *p* > 0.05 (not significant, n.s.). Error bars in all graphs represent standard deviation.

## 5. Conclusions

In summary, our study has demonstrated that canthin-6-one could be an alternative drug candidate for cancer to the current anti-angiogenic therapeutics which mainly targets the VEGFA/VEGFR2 pathway [7]. Our study has also revealed that zebrafish phenotypic screens can be used to identify potential anti-angiogenic agents that work independent of the VEGFA/VEGFR2 pathway and yet synergises with VEGFR inhibitors to block angiogenesis. Further studies should aim to determine the mechanism of action of canthin-6-one and to enhance the efficacy and selectiveness of canthin-6-one through structural activity relationship studies. Apart from cancer, future studies should also investigate whether canthin-6-one can be used to treat other human diseases with excessive angiogenesis such as ocular diseases [49] and vascular anomalies [50].

## Data Availability

Data is contained within the article and Supplementary Material.

## Supplementary Materials

The following supporting information can be downloaded at: https://www.mdpi.com/article/10.3390/ph17010108/s1: Figure S1: Canthin-6-one, kaempferol, and ayanin at 20 µM inhibits developmental angiogenesis in zebrafish. Figure S2: Canthin-6-one impairs HUVEC viability and proliferation. Figure S3: Canthin-6-one does not induce endothelial cell apoptosis. Figure S4: Original western blot images of Figure S3A. Figure S5: Canthin-6-one does not inhibit VEGFA-induced phosphorylation of VEGFR2 in HUVECs and *vegfa*/*vegfr2* expression in zebrafish. Figure S6: Original western blot images of Figure 3E. Figure S7: Canthin-6-one does not bind efficiently with VEGFR2.
