# Supplementary material for: Canthin-6-One Inhibits Developmental and Tumour-Associated Angiogenesis in Zebrafish
Source: Pharmaceuticals (Basel). 2024 Jan 12;17(1):108. doi: 10.3390/ph17010108 (PMC10819238; doi:10.3390/ph17010108)
Supplement: Supplementary file 1 [file pharmaceuticals-17-00108-s001.zip › pharmaceuticals-2751363-supplementary.pdf]

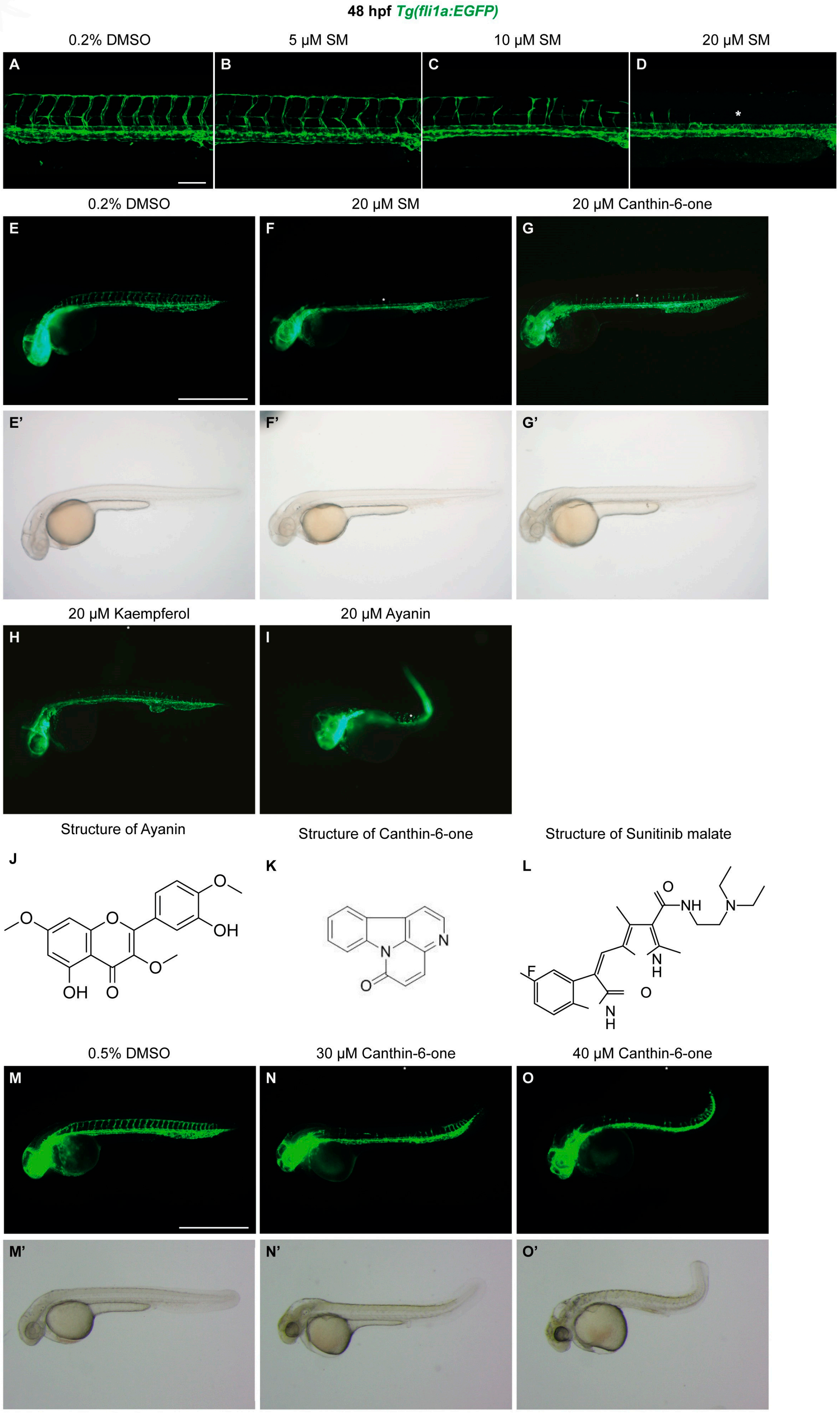
48 hpf Tg(fli1a:EGFP)
0.2% DMSO
5 µM SM
10 µM SM
20 µM SM
A
B
C
D
0.2% DMSO
20 µM SM
20 µM Canthin-6-one
E
F
G
E'
F'
G'
20 µM Kaempferol
20 µM Ayanin
H
I
Structure of Ayanin
Structure of Canthin-6-one
Structure of Sunitinib malate
J
K
L
0.5% DMSO
30 µM Canthin-6-one
40 µM Canthin-6-one
M
N
O
M'
N'
O'

Figure S1. Canthin-6-one, kaempferol, and ayanin at 20 μM inhibit developmental angiogenesis in zebrafish. (**A–D**) Lateral confocal images of 48 hpf *Tg(fli1a:EGFP)* embryos treated with 0.2% DMSO (n = 16, **A**) or sunitinib malate (SM) at either 5 μM (n = 17, **B**), 10 μM (n = 15, **C**), or 20 μM (n=20, **D**). Only SM at 20 μM completely inhibits ISV development. (**E–I**) Lateral fluorescent (**E–I**) and trans-light images (**E′–G′**) of 48 hpf *Tg(fli1a:EGFP)* embryos treated with either 0.2% DMSO (**E,E′**), 20 μM sunitinib malate (SM, **F,F′**), 20 μM canthin-6-one (**G,G′**), 20 μM kaempferol (**H**), or 20 μM ayanin (**I**). White asterisks indicate lack of intersegmental vessels (ISVs). (**J–L**) Structure of ayanin (**J**), canthin-6-one (**K**), or SM (**L**). (**M–O′**) Lateral fluorescent (**M-O**) and trans-light images (**M′–O′**) of 48 hpf *Tg(fli1a:EGFP)* embryos treated with 0.5% DMSO (n = 20, **M,M′**) or canthin-6-one at either 30 μM (n=17, N,N′) or 40 μM (n=18, O,O′). Scale Bars: 100 μm for (A) and 1 mm for (E) and (M).

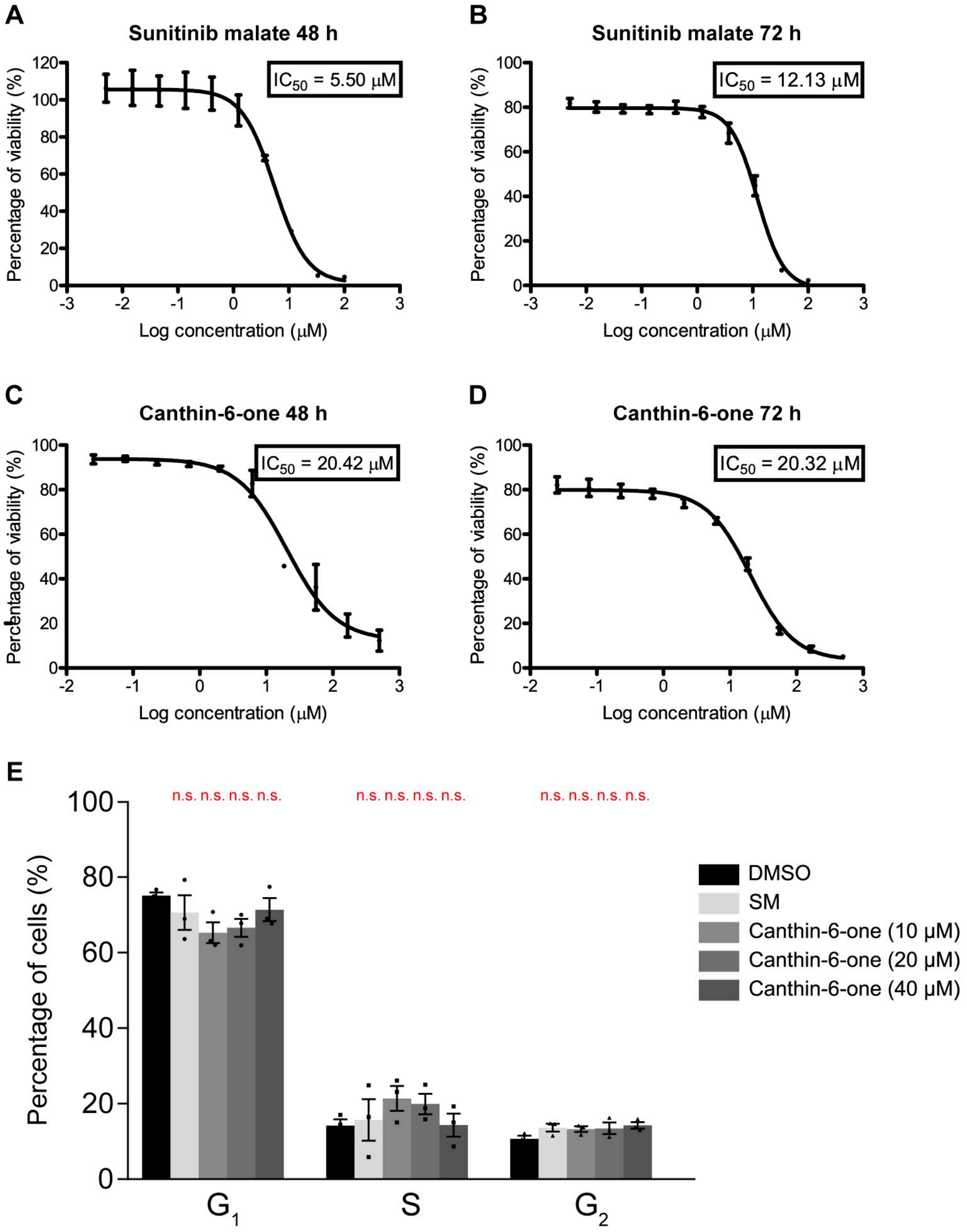

Figure S2. Canthin-6-one impairs HUVEC viability and proliferation. (**A-D**) Cell viability plot of HUVECs treated with either sunitinib malate (SM, A,B) or canthin-6-one (**C,D**) at 0.05 to 1000 μM for 48 h (**A,C**) or 72 h (**B,D**, n = 3). (**E**) Bar chart showing percent-age of HUVECs treated with either DMSO, SM, or canthin-6-one at indicated concentrations in G0/G1, S, or G2/M phase (n = 3). While not significant, canthin-6-one treatment at all concentrations showed a marginal increase of percentage of HUVECs in G2/M phase. Statistical test: Kruskal-Wallis test was conducted for graph **(E)**. p > 0.05 (not significant, n.s.).

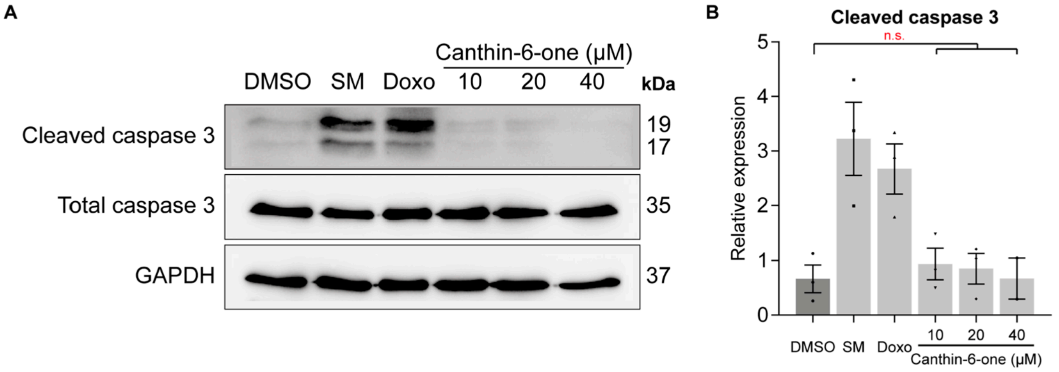

Figure S3. Canthin-6-one does induce endothelial cell apoptosis. (**A**) Western blot analysis of lysates isolated from HUVECs treated with either 0.2% DMSO, 10 μM sunitinib malate (SM), 500 nM doxorubicin, or canthin-6-one at indicated concentrations for 1 h (n = 3). Protein levels of cleaved caspase 3, total caspase 3, and GAPDH were assessed. The full-length blots are presented in Figure S4. (**B**) Densitometry analysis of cleaved caspase 3 protein levels from western blot analysis in image (**A**). Cleaved caspase 3 protein level was normalised to total caspase 3 protein level. Statistical test: Kruskal-Wallis test was conducted for graph **(B)**. $p > 0.05$ (not signifi-cant, n.s.).

Cleaved caspase 3

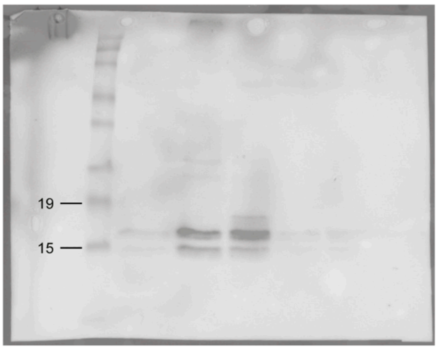

Total caspase 3

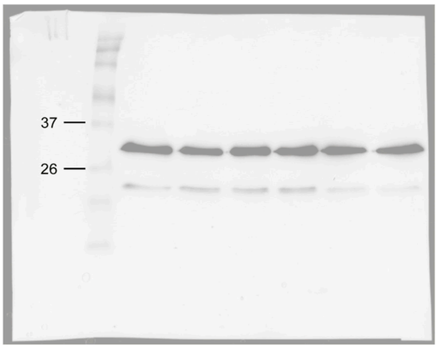

GAPDH

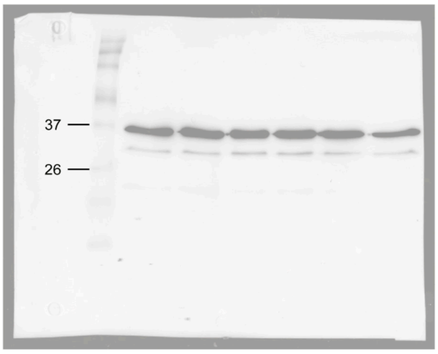

Figure S4. Original western blot images of Figure S3A. Images were taken using the Bio-Rad ChemiDoc Imaging system (Bio-Rad, CA, USA). Original western blot images were processed using the Fiji image processing software.

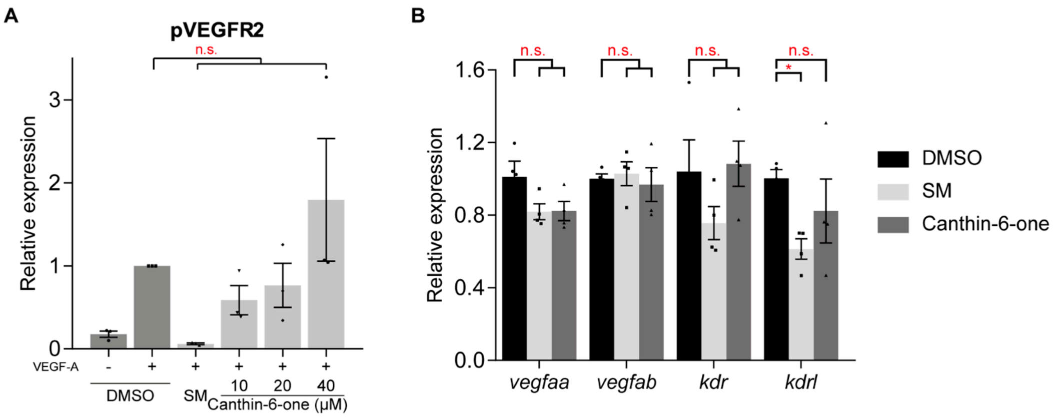

Figure S5. Canthin-6-one does not inhibit VEGFA-induced phosphorylation of VEGFR2 in HUVECs and *vegfa/vegfr2* expression in zebrafish. (**A**) Densitometry analysis of pVEGFR2 protein levels from western blot analysis in Figure 3E. pVEGFR2 protein level was normalised to total VEGFR2 protein level. (**B**) qPCR analysis of expression levels of *vegfaa*, *vegfab*, *kdr*, or *kdrl* in 48 hpf zebrafish embryos treated with either 0.2% DMSO, 20 μM SM, or 20 μM canthin-6-one from 16 hpf (n = 3). All data were normalised to *actb1* mRNA level. Statistical test: Kruskal-Wallis test was conducted for graphs (**A,B**). *p* £ 0.05 (*), *p* > 0.05 (not significant, n.s.).

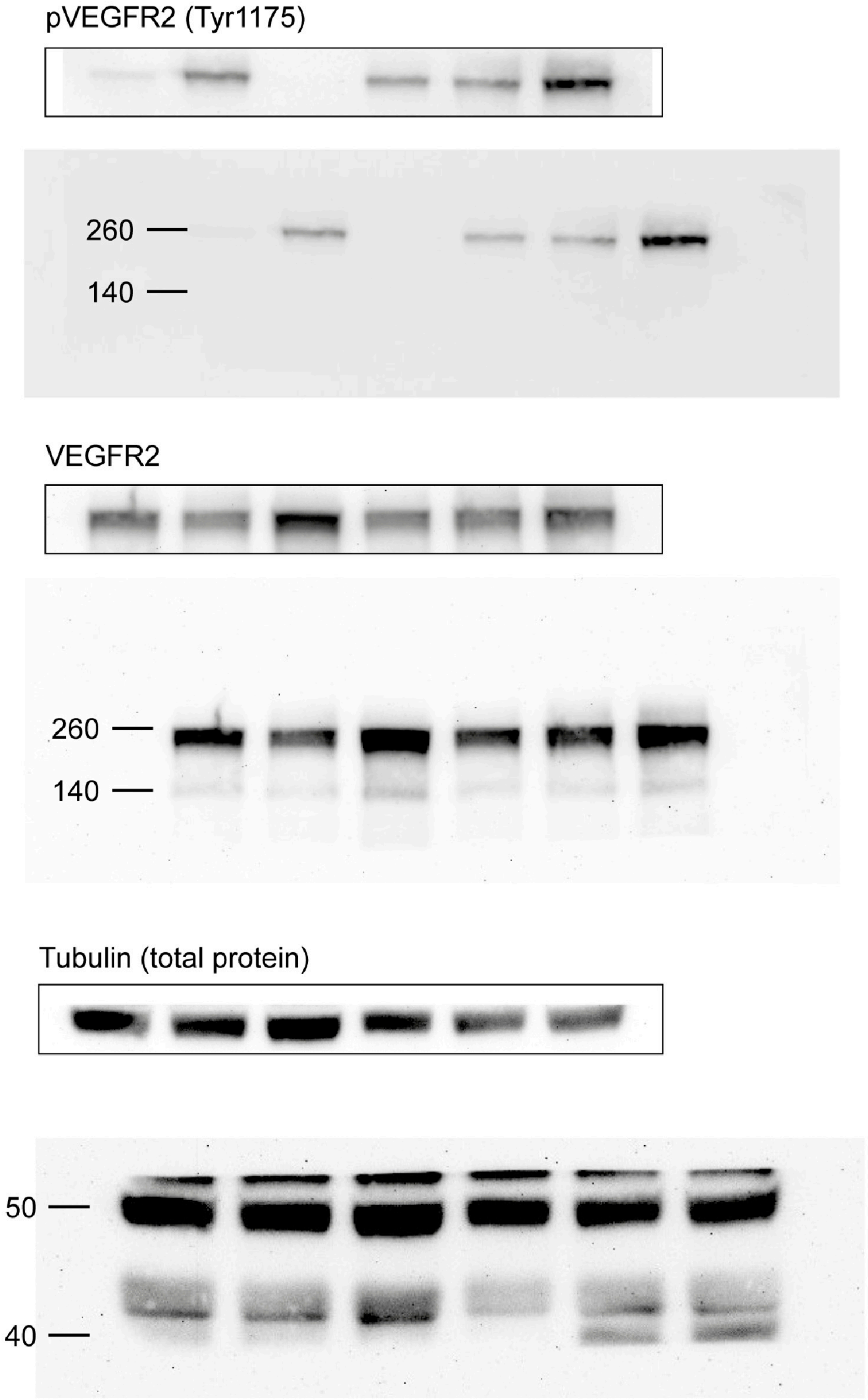

Figure S6. Original western blot images of Figure 3E. Images were taken using the ChemilmagerTM imaging system (Alpha Innotech, CA, USA). Original western blot images were processed using the Fiji image processing software.

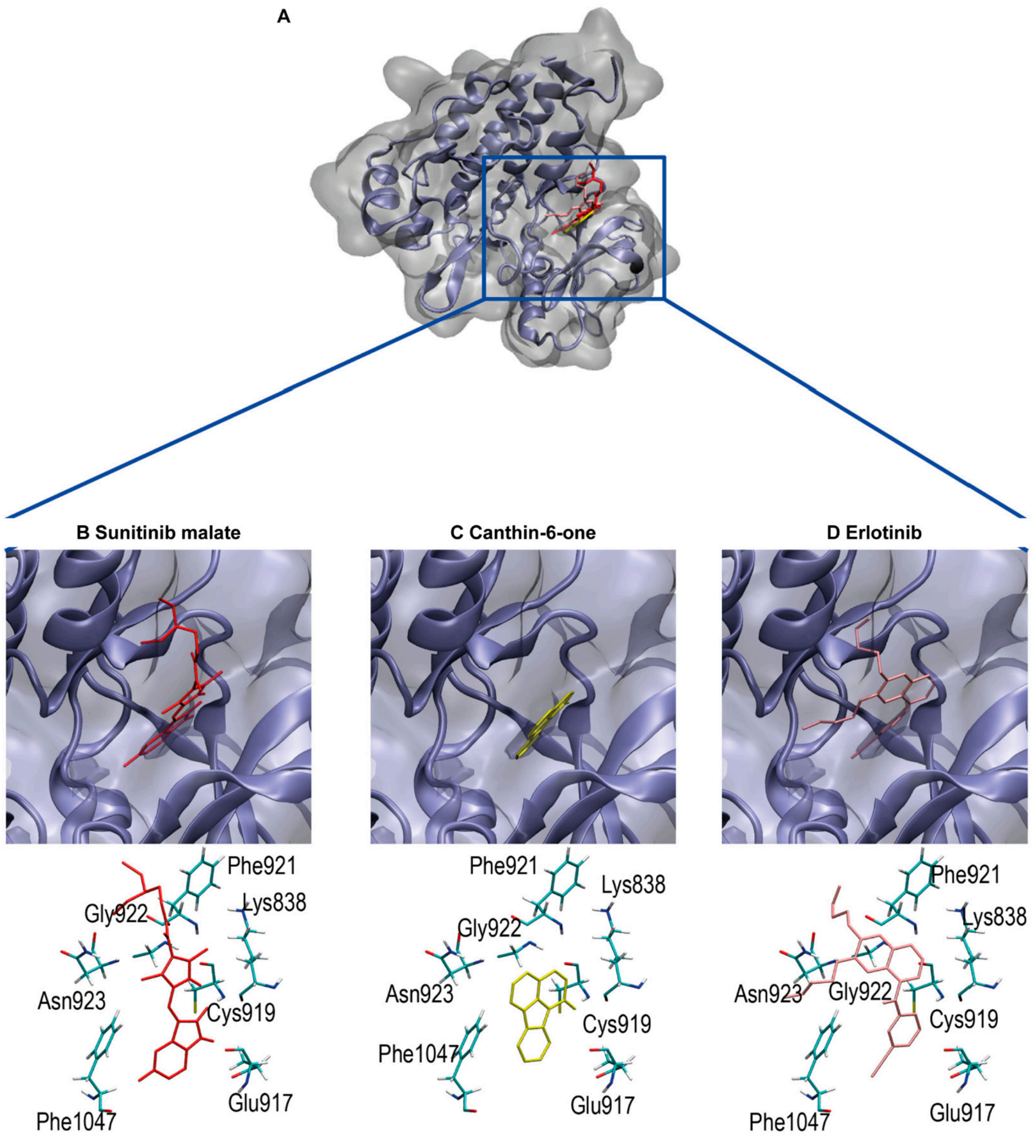

Figure S7. Canthin-6-one does not bind efficiently with VEGFR2. (**A-D**) Potential interacting residues at the binding sites of VEGFR2 (**A**) with sunitinib malate (SM, red, **B**), canthin-6-one (yellow, **C**), or erlotinib (pink, **D**).
